# PGC1α: Friend or Foe in Cancer?

**DOI:** 10.3390/genes9010048

**Published:** 2018-01-22

**Authors:** Francesca Mastropasqua, Giulia Girolimetti, Maria Shoshan

**Affiliations:** 1 Department of Oncology-Pathology, Karolinska Institute, 171 76 Stockholm, Sweden; francesca.mastropasqua@gmail.com; 2 Department of Medical and Surgical Sciences (DIMEC), Unit of Medical Genetics, University of Bologna, 40138 Bologna, Italy; giulsgiuls85@gmail.com

**Keywords:** PGC1α, *PPARGC1A*, tumor progression, tumor cell metabolism

## Abstract

The PGC1 family (Peroxisome proliferator-activated receptor γ (PPARγ) coactivators) of transcriptional coactivators are considered master regulators of mitochondrial biogenesis and function. The PGC1α isoform is expressed especially in metabolically active tissues, such as the liver, kidneys and brain, and responds to energy-demanding situations. Given the altered and highly adaptable metabolism of tumor cells, it is of interest to investigate PGC1α in cancer. Both high and low levels of PGC1α expression have been reported to be associated with cancer and worse prognosis, and PGC1α has been attributed with oncogenic as well as tumor suppressive features. Early in carcinogenesis PGC1α may be downregulated due to a protective anticancer role, and low levels likely reflect a glycolytic phenotype. We suggest mechanisms of PGC1α downregulation and how these might be connected to the increased cancer risk that obesity is now known to entail. Later in tumor progression PGC1α is often upregulated and is reported to contribute to increased lipid and fatty acid metabolism and/or a tumor cell phenotype with an overall metabolic plasticity that likely supports drug resistance as well as metastasis. We conclude that in cancer PGC1α is neither friend nor foe, but rather the obedient servant reacting to metabolic and environmental cues to benefit the tumor cell.

## 1. Introduction

The PGC1 family (Peroxisome proliferator-activated receptor γ (PPARγ) coactivators) isoforms α and β are transcriptional coactivators generally described as master regulators of mitochondrial biogenesis (mitobiogenesis) and function, including oxidative phosphorylation (OXPHOS), fatty acid/lipid metabolism, and regulation of reactive oxygen species (ROS) levels [1,2]. Of the two isoforms, PGC1α in particular is crucial for the rapid adaptation of cells to energy-demanding situations. It is expressed especially in metabolically active tissues, such as the liver, cardiac and skeletal muscle and kidneys, as well as adipose tissue and the brain [1,2]. Given the biological consequences of the altered and highly adaptable metabolism of tumor cells, and the molecular complexity of all types of cancer, it is of interest to investigate PGC1α in cancer. Considering the central role of various mitochondrial functions in cancer, and the wealth of studies on PGC1α in other diseases, it is somewhat surprising that there is so little on PGC1α and cancer. Many reports in the literature appear contradictory, in that both high and low levels of PGC1α expression have been reported to be associated with cancer and worse prognosis, and in that PGC1α has been attributed with oncogenic as well as tumor suppressive features. Although other members of the PGC1 family, or PGC1β and PGC-1-related coactivator (PRC), are likely relevant for the overall understanding of cancer cell metabolism, we will in the interest of stringency focus on PGC1α. Here, we will first briefly review the roles of PGC1α in healthy tissue and disease, and then findings related to cancer, in particular carcinomas. Due to a dearth of studies, discussion of these diverse findings must, in some cases, include associative rather than proven causalities and connections, and we would therefore like to present this review as a map of interesting uncharted areas for future research. 

### 1.1. The Gene and the Protein

The Peroxisome proliferator-activated receptor γ co-activator 1 α (*PPARGC1A*) gene maps to chromosome 4p15.2 and is composed of 13 exons coding for a 91 kDa protein. The N-terminal region of the protein recruits and interacts with histone acetyl transferase proteins (e.g., p300 or SRC-1) to promote access of the transcriptional complex to the DNA. The C-terminal region interacts directly with the transcription partners, but can also interact with the splicing machinery processing the nascent mRNAs, and thus further complicating its potential as transcriptional regulator [3]. 

The PGC1α protein partners with about twenty nuclear factors, such as PPARγ, nuclear respiratory factor 1-2 (Nrf1-2), Forkhead box O3 (FoxO3a), cyclic-AMP (cAMP) response element-binding protein (CREB) and estrogen related receptor-α (ERRα) [1,2,4]. PPARγ is well-known for its roles in adipogenesis, thermogenesis and mitobiogenesis, whereas Nrf1-2, ERRα and FoxO3a are of importance for mitobiogenesis, antioxidant defense and rapid responses to metabolic stress; importantly, they are targets as well as partners of PGC1α [5,6].

Alternative promoters and alternative splicing allows the expression of several isoforms that are either tissue-specific and/or induced upon specific kinds of stress; an excellent review of the isoforms is provided in [3]. Briefly, most of them are involved in mitochondrial programs, whereas PGC1α is associated with vascular endothelial growth factor (VEGF) expression in skeletal muscle undergoing extended exercise, and PGC1α4 has unique effects on muscle hypertrophy. Differential structures of the isoforms affect how they are posttranslationally modified and also their responses to stimuli such as cAMP and glucocorticoids. The truncated form NT-PGC1α exposes a nuclear export signal that renders the protein cytosolic under basal conditions, whereas cAMP stimulation of brown adipocytes induces its nuclear localization [3]. 

Being a key regulator of metabolic adaptation, PGC1α activity needs to be finely regulated also at a post-transcriptional level. Inhibition by acetylation by, for example, histone acetyltransferase GCN5, and activation by the sirtuin 1 (SIRT1) deacetylase represent the major cycle of regulation in response to metabolic changes, as these enzymes respond to levels of acetyl-CoA and NAD^+^, respectively; this is reviewed in [7]. Useful overviews of other pathways are provided in [2,3]; briefly, PGC1α activity is promoted by stress sensors such as the kinases AMP-activated protein kinase (AMPK) and p38, whereas phosphorylation by AKT inhibits it. The AMPK-mediated response to low-energy conditions is to promote catabolic processes including glycolysis as well as OXPHOS and fatty acid β-oxidation. Ubiquitin-proteasomal degradation confers a PGC1α half-life of around 20 min; intriguingly, the E3 ligase Fbw7β isoform promotes degradation whereas the Fbw7α nuclear isoform stabilizes the protein [8,9]. 

In addition to a nuclear localization, PGC1α has been found associated with mitochondrial DNA (mtDNA) in complex with its activator, SIRT1 and mitochondrial transcription factor A (TFAM), another downstream target of PGC1α [10] but the significance of this remains unclear [11].

### 1.2. PGC1α in Healthy Tissue and in Non-Cancer Disease 

In the literature, PGC1α is often presented in terms of promoting oxidative metabolism and mitobiogenesis. Examples from healthy tissue include the neonatal heart, in which cardiomyocytes need to switch from a glycolytic to an oxidative metabolism and this switch involves PGC1α [12], and thermogenesis induced by cold in mitochondria-rich brown adipose tissue [1]. PGC1α is also central to antioxidant defense and redox balance, by regulating expression of factors such as Nrf2 and manganese superoxide dismutase (MnSOD), and by promoting NADPH production, and thereby counters increased ROS levels in highly oxidative cells and protects against inflammation [13,14]. Other normal functions of PGC1α include support of hepatic gluconeogenesis; thus, insulin represses PGC1α expression via insulin response sequences in the promoter [15]. PGC1α may also promote β-oxidation of lipids as a means of maintaining energy homeostasis [2].

It is thus not surprising that decreased PGC1α expression leading to dysregulation of mitochondrial functions is of pathophysiological importance. PGC1α downregulation in cardiac muscle by inflammatory cytokines is directly associated with cardiac dysfunction [16]. In both Parkinson´s (PD) and Alzheimer´s disease (AD), brain neurons show decreased respiratory and antioxidant capacity, and impaired mitobiogenesis associated with mtDNA mutations as well as downregulation of PGC1α expression [17]. In PD, downregulation may be a result of non-canonical PGC1α promoter methylation [18], and/or loss of antioxidant function due to lack of the E3 ubiquitin ligase Parkin leading to accumulation of Parkin-interacting substrate (PARIS; zinc finger protein 746, (ZNF746)), a transcriptional repressor of PGC1α [19]. Conversely, overexpression of PGC1α can also be deleterious, as indicated in a rat model with prolonged local overexpression of PGC1α leading to degeneration of dopaminergic neurons [20]. Considerable research on PGC1α in diabetes type 2 (DM2) has shown that although its high expression is beneficial in certain tissues it is deleterious in others, rendering PGC1α difficult to target pharmaceutically [21]. 

Of interest for the present topic is the epidemiological as well as etiological association between cancer and DM2 and/or obesity [22]. It is well known that PGC1α and other proteins involved in mitochondrial respiration are down-regulated in insulin-resistant muscle [23,24]. The underlying mechanisms include promoter hypermethylation [25] that can be due to effects of saturated fatty acids [18,26], and of inflammatory cytokines such as tumor necrosis factor-like inducer of apoptosis (TWEAK) of the tumor necrosis factor (TNF) family [27]. Interest in the influence of such pathways on tumor cells and tumor stromal cells is growing.

## 2. PGC1α in Cancer

### 2.1. Low and High Expression of PGC1α in Cancer 

**Figure 1:**
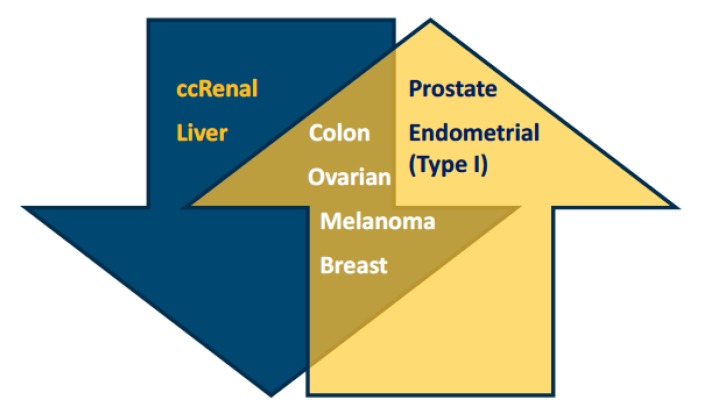
Peroxisome proliferator-activated receptor γ isoform α (PGC1α) levels are not dependent on cancer type. Studies have reported different levels of PGC1α in the same cancer type, and to the best of our knowledge, PGC1α high or low expression is not a characteristic of a certain type of cancer from a specific organ or tissues. The blue arrow lists cancers reported to express low levels of PGC1α: renal (clear-cell) [29], liver [30,31]. The yellow arrow lists cancers reported to express high levels of PGC1α: prostate [32], endometrial [33]. The intersection of the two arrows lists cancer biopsies or cell lines for which both low and high levels of PGC1α have been reported: colon [34,35,36], ovarian [37,38], melanoma [39,40,41], breast [42,43,44,45,46].

Although about a dozen PGC1α alternative transcripts have been reported in healthy tissue [3], no specific variant or isoform has to our knowledge been addressed in cancer studies. Increased as well as decreased levels of PGC1α have been observed in a range of cancer types, and both cases have also been associated with worse prognosis [2,28]. Even within one cancer type, such as breast cancer, reports differ regarding PGC1α levels. This subsection provides examples of low and high expression of PGC1α in cancer that are summed up in Figure 1, and which will be further discussed below. 

Low-level expression and correlation with worse outcome have been reported in studies on breast and liver carcinomas [30,42,43]. Melanomas harboring constitutively active BRAF protein, or about half of all melanoma cases, showed low levels of PGC1α due to BRAF suppression of the melanocyte lineage factor, MITF, which directly regulates PGC1α [39,40]. Intestinal adenomas from an *Apc*^−/−^ mouse model as well as from familial adenomatous polyposis patients showed PGC1α levels that were less than half of those in adjacent normal mucosa [34]. In a tissue microarray of ovarian carcinomas, we found that the highly chemoresistant clear-cell subtype was characterized by the lack of expression of both PGC1α and TFAM, unlike the more treatable high-grade serous carcinoma subtype [37]. Interestingly, stabilization of hypoxia-inducible factor 1 alpha (HIF1α) was associated with downregulation of PGC1α TFAM mRNA and protein levels in likewise chemoresistant Von Hippel-Lindau tumor suppressor protein (VHL)-deficient clear-cell renal carcinoma tumors and cell lines [29]. 

High PGC1α expression was found in circulating cancer cells and to support invasivity in a mouse model of breast cancer [44]. Similarly, PGC1α expression was enriched in breast cancer metastases in xenografts and in patients [45]. In breast cancer patients, plasma concentrations of PGC1α were higher than in healthy controls and multivariate analysis showed a correlation between high PGC1α and worse prognosis [46]. In a study on prostate cancer, androgens signaling via AMPK led to increased PGC1α mitobiogenesis and OXPHOS but also glycolysis; analyses of mouse xenografts and patient samples suggested that AMPK/PGC1α correlated with cancer growth [32]. Oncocytomas, e.g. thyroid, are generally less aggressive than their non-oncocytic counterparts. They are associated with pathogenic mtDNA mutations leading to dysregulated Complex I, and show mitochondrial hyperplasia and upregulation of PGC1α [47,48]. Compared to normal tissue, endometrial carcinomas type I also showed upregulation of PGC1α, and an oncocytoma-like phenotype [33], and in a later report also upregulation of mtDNA content and citrate synthase activity, but no association with stage, grade or invasivity [49]. Also, similar to oncocytomas, type I endometrial cancer harbored pathogenic mtDNA mutations and aberrant Complex I [50]. Any comparison in these respects with the more aggressive type II cancer has to our knowledge not been published. A possible connection between the indicated mtDNA mutations and a compensatory upregulation of PGC1α and mitobiogenesis has been hypothesized [51] but not yet proven. 

### 2.2. PGC1α and Oncogenicity 

No genetic lesions conferring constitutive or abolished expression of PGC1α have as yet been identified in cancer, although some gene variants may influence cancer risk. A multicenter study on around 3000 single nucleotide polymorphisms (SNPs) and risk for ovarian cancer suggested in 2011 that variants in genes involved in mitobiogenesis, including *PPARGC1A*, may influence susceptibility to ovarian cancer [52]. Similarly, in a case-control study on 701 colorectal cancer patients, a polymorphism (rs3774921) in the *PPARGC1A* gene was associated with a higher risk of colorectal cancer, and more so in combination with a pro-inflammatory diet [53]. As of November 7, 2017, there were 302 *PPARGC1A* mutations annotated in the Catalogue of Somatic Mutations in Cancer (COSMIC) database (v83), amounting to a proportion of about 0.007% of all analyzed samples. The Functional Analysis through Hidden Markov Models (FATHMM-MLK) algorithm (http://fathmm.biocompute.org.uk/) predicts functional and phenotypic effects of missense variants; based on a FATHMM score ≥0.7, it was predicted that a majority of the mutations may be pathogenic [54]. However, these predictions need experimental verification.

It is now clear that oncogenic signaling and proliferation confer greatly increased anabolic demands on the cancer cell, and that cancer cell metabolism therefore goes far beyond the Warburg effect, that is, the high dependence on aerobic glycolysis and downregulation of mitochondrial respiration [55]. The observed upregulation of respiration, upregulated glutamine and lipid metabolism, altered redox status and tricarboxylic acid (TCA) cycle function, and so on, all involve high mitochondrial content and activity [55,56] and suggest that PGC1α expression would be high in cancer. However, as seen above, there is no such clear correlation.

In line with a lack of obvious correlation, the influence of well-known oncogenic signaling pathways on PGC1α expression does not appear to be direct nor constitutive. The general lack of direct oncogenic influence is in agreement with PGC1α being more sensitive to metabolic cues, in terms of regulation of both expression and activity. However, there are some exceptions:

- *BRAF*: Constitutively activated BRAF, commonly seen in melanomas, was shown to suppress the melanocyte lineage factor MITF, leading to loss of MITF-regulated PGC1α and upregulation of a glycolytic metabolism [39].
- *p53*: Experiments on cancer cell lines showed that PGC1α can bind to and potentiate p53 transactivation of cell cycle arrest and metabolic genes. Furthermore, glucose starvation and abrogation of PGC1α led to ROS overload and apoptosis [57]. It was also found that in PGC1α proficient cells, prolonged starvation led to PGC1α degradation by the ubiquitin-proteasome pathway and apoptosis [57]. However, we have not observed a similar sensitivity to starvation in an ovarian cancer cell line devoid of both p53 and PGC1α expression [37], indicating that cancer cells may develop compensating mechanisms. Conversely, in a mouse model and in samples from chronic lymphocytic leukemia patients, loss of p53 through deletion of chr17p correlated with increased expression of PGC1α and its downstream effector TFAM, and with increased mitochondrial respiratory activity [58], although no clear causality was demonstrated. Inverse correlations between p53 and PGC1α have been observed also in non-cancer contexts, for example, upregulation of p53 due to telomere dysfunction repressed both PGC1α and PGC1β [59]; such mechanisms may turn out to be of interest in the cancer field.
- *MYC*: An inverse relationship between MYC and PGC1α has been demonstrated in cardiac myocytes [60] as well as in pancreatic cancer stem cells [61]. The latter study also showed that PGC1α/MYC ratios represent a spectrum of tumor-promoting metabolic phenotypes ranging from OXPHOS-based to glycolytic. As MYC regulates glucose and glutamine metabolism and also mitobiogenesis in cancer cells [62], this might together with MYC-dependent PGC1β expression [63] explain why PGC1α negative tumor cells nevertheless have functioning mitochondria and metabolism. Although not formally shown, it is also conceivable that the MYC/PGC1α ratio can be regulated by levels of the transcription factor FoxO3a, since this is a direct transcriptional regulator of PGC1α [6] and is in metabolic contexts also a negative regulator of MYC [64]. Similar to PGC1α, both high and low expression of FoxO3a has been associated with cancer and worse prognosis, in line with the notion that metabolic plasticity is central to tumor progression and treatment resistance.
- *ERRα* Although not oncogenic as such, the role of the estrogen-related receptor (ERR) family should not be overlooked in cancer cells expressing PGC1α. Like the other members of the ERR family, ERRα does not bind estrogens and their transcriptional activities are ligand-independent. Indeed, PGC1α β act as surrogate ligands for ERRα and the resulting PGC1/ERRα axis is of importance in cancer and cancer cell metabolism [28,65]. Similar to PGC1α, ERRα is required for rapid stress responses but less so for basal energy regulation. It binds to promoters of most enzymes in glucose, glutamate and fatty acid metabolism and the TCA cycle, and is upregulated in many cancers and associated with unfavorable outcomes [65]. Interestingly, there are reports on ERRα inhibitors inhibiting the growth of PGC1α proficient cells [2,41]. In order to help clarify the roles of PGC1α and its different partners and pathways, future studies should address for instance the prognostic significance of the combined PGC1α ERRα.

### 2.3. Mechanisms of Regulation of PGC1α Levels in Cancer

The two major inducers of increased expression of PGC1α are AMPK-mediated phosphorylation of PGC1α required for the PGC1α autoinduction of its own promoter [66], and the PGC1α ERRα auto-coactivation system [67]. Other promoting factors include p53 and the melanoma lineage factor MITF [2]. By contrast, there are numerous mechanisms of downregulation of PGC1α protein levels. Firstly, the *PPARGC1A* gene is prone to hypermethylation, as evidenced in studies on other diseases, particularly metabolic syndrome conditions [18,25,68]. Methylation can in diabetic subjects be due to the DNA methyltransferase DNMT3b [25], which is normally involved in DNA methylation during embryogenesis and which was found upregulated in breast, colon and prostate cancers [69]. The role of *PPARGC1A* methylation in cancer has not been studied. 

Secondly, PGC1α may be subject to degradation via the ubiquitin-proteasome pathway. Phosphorylation of the PGC1α protein by GSK3β marks it for degradation, and may at least in non-cancer cells occur in response to oxidative stress [70]. In neurons and cultured cancer cells the nuclear protein necdin inhibited ubiquitinylation and degradation of PGC1α and thereby helped maintain OXPHOS integrity [71]. Interestingly, necdin suppressed metastasis in breast cancer [72], and has shown tumor suppressive features in other cancers [73,74]. Thirdly, factors such as TGF-β suppress PGC1α expression in diabetic muscle [75], and in lung cancer cells in vitro [76]. Other inflammatory cytokines that are known to influence tumorigenesis, such as TNFα IL-6 and TWEAK, may also suppress PGC1α expression [77,78,79]. More research is warranted to investigate the role of all these, and similar, factors in regulation of PGC1α and metabolism in cancer cells.

A fourth mechanism involves mitophagy which helps remove dysfunctional mitochondria, and which is mediated by the E3 ligase Parkin. Loss of Parkin activity results in accumulation of one of its targets, PARIS (ZNF746), which is known to act as a transcriptional repressor of PGC1α [19]. As many reports show deletion or inactivating mutations of Parkin in several types of cancer [80], it would be interesting to examine PARIS and downregulation of PGC1α expression in these. 

Fifthly, about two dozen microRNAs have been found to downregulate PGC1α, and the majority of these findings are in normal tissue, typically skeletal muscle and hepatocytes. For instance, increased levels of circulating miR-130b were observed in obese patients and found to directly downregulate PGC1α in muscle [81]. Regarding cancer, the micro RNAs (miRNAs) 485, 485-3p and -5p were shown to directly inhibit expression of PGC1α and to be downregulated in 30 breast cancer patient samples, and significantly more so in the metastatic cases [82]. Based on a mouse hepatocarcinoma model and human hepatocarcinoma cell lines, PGC1α was reported to be directly downregulated by miR-23a, which in turn was upregulated by IL6/signal transducer and activator of transcription 3 (STAT3) signaling [83]. Many other studies have reported roles for miR-23a in cancer without investigating PGC1α; interestingly, this miR is often downregulated in tumors [84]. Similarly, miR-217, which inhibits proliferation and induces apoptosis, was found to bind to PGC1α mRNA in breast cancer cell lines and downregulate its expression [85]. Together, these as yet scant reports on miRNAs and PGC1α reinforce the notion that PGC1α expression levels in cancer may vary greatly. 

Finally, reduced expression is not the only means of the cancer cell to prevent PGC1α activity, as it can also be inhibited by acetylation, notably by GCN5. This acetyltransferase is overexpressed in hematopoietic malignancies [86] and in several types of carcinoma, e.g., colon cancer, where MYC was found to promote its expression [87], thus strengthening an inverse relationship between MYC and PGC1α. 

## 3. Pro-Oncogenic or Tumor Suppressive PGC1α in Tumorigenesis and Progression? 

### 3.1. A Model 

**Figure 2:**
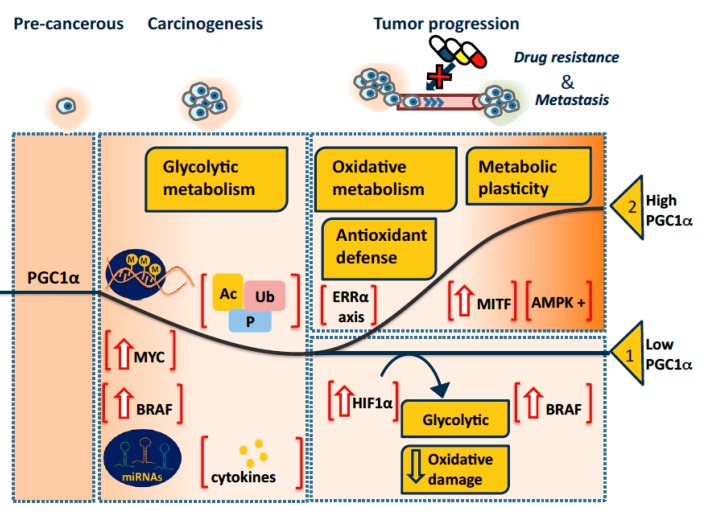
Schematic overview of PGC1α levels in cancer during carcinogenesis, metastasis and acquisition of chemoresistance. This model proposes that carcinogenesis may in some tissues entail loss of a tumor-suppressive function of PGC1α, and in many tissues also a switch to a glycolytic cell metabolism. PGC1α downregulation may be due to promoter methylation or ubiquitinylation of the PGC1α protein; both mechanisms are seen also in non-cancer cells [25,70]. Transcriptional repression may occur via high levels of MYC [60,61], active BRAF [39], or hypoxia-induced factor 1α (HIF1α) [29], miRNA regulation [82,83,84,85], or cytokine effects [75,76,77,78,79]. Inactivation of the PGC1α protein is also possible, notably by acetylation and phosphorylation [1,86]. Tumor progression, i.e., chemoresistance and metastasis, is proposed to proceed either in a PGC1α-low (“option 1” in the figure), or in a PGC1α-high context (“option 2”). Tumors can maintain low PGC1α expression due to the mechanisms outlined above, while in “option 2” PGC1α levels may be high due to regulators such as ERRα, the main autoregulatory transactivation partner of PGC1α [67], melanoma lineage factor (MITF) [39] or activated AMP-activated protein kinase (AMPK) [66]. Tumor progression is supported by development of a metabolic plasticity that allows rapid adaptation to the microenvironment and nutrient availability. PGC1α is often associated with such plasticity. *Abbreviations*: M: methylation; Ac: acetylation; Ub: ubiquitinylation; P: phosphorylation.

Seeking to summarize and reconcile the apparently contradictory roles of high and low PGC1α levels, we here propose a hypothetical model of its roles in tumorigenesis and tumor progression (Figure 2) and provide supporting examples. 

#### 3.1.1. PGC1α in Tumorigenesis

We first consider PGC1α in initial tumorigenesis and reports suggest that it may at least in some tissues have a tumor-suppressive role. Downregulation of PGC1α mRNA and/or protein levels as part of cancer development was seen in intestinal [34,35] and liver cancer [31,35]. In [34], the tumor suppressive function of PGC1α was clearly to support the differentiation of proliferating crypt cells into enterocytes with higher respiratory capacity and the subsequent apoptosis. Interestingly, a role for PGC1α in preventing metastasis in melanomas has been shown and is described in Section 3.1.2. As many types of cancer are age-related diseases, the fact that PGC1α activity and mitochondrial function decrease with age [88,89] might also reflect a tumor-suppressive role in tumorigenesis. Along similar lines, exercise and a calorie-restriction diet are known to promote both PGC1α activity and healthy aging [90]. Overall, in line with the cancer-preventive effects of various antioxidant agents, the ability of PGC1α to upregulate antioxidant defense is likely part of its tumor-suppressive capacity. Oxidative DNA damage is known to contribute to carcinogenesis, and the roles of antioxidant enzymes regulated by PGC1α, such as MnSOD and Nrf2, in preventing such damage have been addressed in a number of studies. However, PGC1α and DNA damage protection in carcinogenesis has not been studied.

Downregulation of PGC1α activity could be due to, for example, hypermethylation of the *PPARGC1A* gene, dysregulation of ubiquitin-proteasomal degradation or to effects of inflammatory cytokines. In a PGC1α low cancer cell, the oncogenic driver might for instance be activated BRAF [39], or MYC, based on an inverse relationship between MYC and PGC1α [60,61]. Moreover, loss of PGC1α activity leads to loss of antioxidant defense, such as SOD2 [5], leading to ROS-mediated stabilization of HIF1α and subsequent upregulation of tumor-promoting glucose and glutamine metabolism [91]. 

#### 3.1.2. PGC1α in Tumor Progression

After establishment of cancer in situ, the role of PGC1α in further steps of tumor progression is suggested to be determined by the microenvironmental and metabolic context of the tumor. Thus, the tissue of origin, hypoxia, interactions with non-cancer cells in the stroma, including growth factors, cytokines and catabolites, may all contribute to the metabolic demands of the tumor, as outlined in [92], and hence also to determining whether PGC1α and an oxidative metabolism is advantageous or not for the tumor and tumor progression. This is exemplified by decreased as well as increased OXPHOS activity being associated with metastasis [93]. 

That tumor growth and chemoresistance may develop in the absence of PGC1α expression can be seen in clear-cell carcinomas of both ovarian as well as renal cancer [29,37]. The loss of the VHL tumor suppressor in renal carcinomas is well known to lead to increased stabilization HIF1α and a glycolytic metabolism. HIF1α stabilization was in turn shown to lead to loss of PGC1α expression, and when PGC1α was restored by transfection, respiration and sensitivity to doxorubicin were also restored [29,37]. Moreover, in melanomas, low PGC1α correlated with vertical or invasive growth [41]. A role of PGC1α in preventing melanoma metastasis was shown with treatment with a BRAF inhibitor, which upregulated PGC1α and its downstream target DNA-binding protein inhibitor-2 (ID-2), which in turn led to suppression of metastasis-related genes [41]. The paper points out that this anti-metastatic effect of PGC1α is distinct from its bioenergetic functions [41]. As this adds more complexity to understanding the tumor biological significance of PGC1α levels, it also highlights a need to consider PGC1α as a master regulator of not only mitochondrial function and therefore to map PGC1α transcriptional partners and downstream gene activation in more varied cellular contexts. 

The other ”option” (Figure 2) during tumor progression is that the tumor restores or even overexpresses PGC1α. High PGC1α levels were associated with doxorubicin resistance in melanomas, likely due to increased antioxidant defense [40], and with chemoresistance-promoting proteins MDR1 and ABCG2 in an ovarian cancer cell line made to overexpress PGC1α [38]. However, since the mechanism of action of doxorubicin is well known to involve ROS, and as PGC1α sensitized renal cancer cells to this drug [29], the effects of PGC1α on chemoresistance appear to be more complex, and it would be interesting to compare PGC1α induced antioxidant defense in [29,40].

PGC1α OXPHOS metabolism has in several studies been linked to tumor progression. Bioenergetic screening of 25 ovarian cancer cell lines revealed that while chemosensitive non-metastatic ones were mostly glycolytic, the chemoresistant metastatic cell lines showed metabolically highly active phenotypes with both glycolysis and increased OXPHOS metabolism [36]. Four cell lines were then selected for more detailed study, and the two chemoresistant metastatic ones showed higher expression of PGC1α, the significance of which was supported by increased TFAM and CoxVb expression, and fatty acid β-oxidation [36]. Similarly, a colon cancer circulating cancer cell (CTC) cell line isolated from a patient also showed significantly increased PGC1α expression compared to a non-metastatic colon cancer cell line and other cell lines in the Affymetrix public database [94]. In line with [36], the ensuing pathway analyses identified fatty acid metabolism rather than OXPHOS as the most likely metabolic alteration in these CTCs [94]. Indeed, the PGC1α downstream effectors β-oxidation and fatty acid/lipid metabolism are known to promote metastasis [93]. A functional study on murine as well as human breast cancer cell lines in vitro and in a murine model showed that PGC1α was overexpressed in metastatic cells compared to non-metastatic, and that expression was highest in lung metastatic cells [45]. The same study also created a PGC1α gene signature based on a breast cancer cell line in which PGC1α was knocked down using small interfering RNA (siRNA). The signature was then applied to a gene expression dataset on breast cancer metastases, and was found to be enriched in lung and bone metastases [45]. It is unclear if this reflects an ability of in particular PGC1α-expressing cells to colonize the lungs, or if it reflects a feature of the lung tumor microenvironment to stimulate PGC1α expression promoting further growth. A partial answer might be perceived in the fact that although only a fraction of CTCs actually form metastases, high expression of PGC1α and oxidative metabolism were observed in a mouse model of breast cancer CTCs, and manipulation of PGC1α expression in cell lines in vitro was found to promote OXPHOS, invasion and metastasis [44]. 

Upregulation of a PGC1α/Nrf-1 signature was demonstrated in breast cancer tumor cells relative to stromal cells (n = 28) using laser microdissection; this signature was then confirmed in >2000 additional cases, and in particular the PGC1α target Nrf-1 correlated with metastasis and recurrence [95]. In another study, metastatic capacity in mouse melanoma cell lines depended on mitochondrial ROS; however, metastasis did not come with altered OXPHOS or glycolysis, but rather with aberrant TCA cycle activity [96], wherefore it would be interesting to examine the PGC1α levels in this experimental system. 

There are not enough expression studies to be able to determine whether up- or downregulation of PGC1α mRNA protein dominates in carcinomas, but up-regulation seems likely, notably as part of development of a metabolic plasticity that contributes to survival under cellular stress [36,93]. Moreover, models of tumor microenvironment heterogeneity hold that tumor stromal fibroblasts and/or hypoxic tumor cells are largely glycolytic and produce lactate for the benefit of oxidative tumor cells [97,98], wherefore it would be of interest to examine tissue compartmentalization of PGC1α. 

Metastatic disease can be considered the endpoint of tumor progression, and the highest metastatic potential is believed to reside in the cancer stem cell, or tumor-initiating cell (TIC), subpopulation whose metabolism is briefly discussed below. 

#### 3.1.3. PGC1α and Tumor-Initiating Cells 

Invasivity/metastasis in carcinomas is associated with epithelial-mesenchymal transition (EMT), a dedifferentiation process which confers motility and other features of invasivity. Disease recurrence is increasingly associated with the presence of tumor-initiating cells (TICs), also referred to as cancer stem cells. These cells are highly chemoresistant, form spheres in stem cell medium, and typically show EMT and expression of TIC markers such as CD44, CD117, and aldehyde dehydrogenase 1A (ALDH1A), and up to a 1000-fold higher ”take”, or tumor formation, when injected into nude mice. 

While the literature holds very little on PGC1α and EMT, there is an increasing interest in PGC1α in the TIC phenotype(s) and metabolism. Initially, TICs were believed to be highly glycolytic, in analogy with normal stem cells that reside in hypoxic niches and show immature mitochondria that only during differentiation upregulate PGC1α to develop cristae and an oxidative metabolism [99]; a parallel is seen also in the intestinal proliferative crypt cells that differentiate into enterocytes along with PGC1α expression and increased oxidative metabolism [34]. Recent reports, however, show that TICs may follow the opposite development. For example, pancreatic cancer TICs showed increased PGC1α expression and high OXPHOS, due to downregulation of MYC [61]. They were also uniquely sensitive to metformin, an inhibitor of Complex I and activator of AMPK, and interestingly, a TIC subset that developed metformin resistance showed increased MYC expression and glycolysis along with decreased PGC1α OXPHOS. Although the authors found that PGC1α maintains the TIC capacity for self-renewal, they also concluded that the PGC1α/MYC ratio determines the metabolic phenotype and that this implies a great range of metabolic heterogeneity in vivo [61]. 

A similarly central role of mitochondria and OXPHOS in regulation of TIC properties was shown in cultured breast cancer cells which can form CD44^+^/CD24^−^ spheres under serum-free, non-adherent conditions. ERRα antagonists are known to inhibit growth in PGC1α proficient cells [2], and in order to investigate the role of mitobiogenesis and metabolism in mammosphere formation by MCF-7 cells, the ERRα antagonist XCT790 was used [100]. Pretreatment of monolayers with XCT790 did reduce subsequent mammosphere formation, and both respiration and glycolysis, as well as stem cell signaling pathways such as Sonic hedgehog and Wnt. Moreover, overexpression of ERRα was necessary and sufficient to promote sphere formation, and overexpression of the Wnt pathway transcription factor FOXM1 also promoted mammosphere formation and upregulation of mitochondrial and metabolic proteins [100]. The study showed a central role for mitochondrial biogenesis and function in mammosphere/breast cancer TIC formation. It would be interesting to investigate PGC1α/ERRα/MYC in this system, and to understand whether TICs differ significantly from other cells regarding regulation of mitochondria. 

Compared to their CD117-negative counterparts, ovarian cancer CD44^+^/CD117^+^ TICs isolated from patient ascites were found to express a third TIC marker, ALDH1A, and to overexpress proteins involved in five major metabolic pathways: OXPHOS, β-oxidation, glucose uptake, the pentose phosphate pathway (PPP) and the TCA cycle [101]. Dependence on these pathways was corroborated using inhibitors and various culture conditions: the CD44^+^/CD117^+^ TICs were uniquely sensitive to inhibitors of respiration/OXPHOS and β-oxidation, whereas they survived glucose deprivation by entering a state of quiescence that depended on OXPHOS. Glucose deprivation also improved sphere formation, in further support of OXPHOS being important for the stemness of TICs. The high glucose uptake was found to be utilized in part by the PPP, yielding NADPH for maintaining redox power, and in part for pyruvate fueling the TCA cycle [101]. These results outline the notion of TICs as a phenotype of extreme metabolic plasticity, which complicates most strategies for eradicating them. While this paper did not investigate PGC1α, a recent report shows that PGC1α mitobiogenesis and stem cell markers, were upregulated in spheres formed by the ovarian cancer cell line PA1 grown in stem cell medium [38]. The authors suggest that the hypoxic and ROS-enriched interior of the spheres caused PGC1α upregulation, since it was blunted by ROS scavengers, and conversely, exogenous ROS (superoxide) induced PGC1α in the 2D cell culture. 

Similar to the CD44^+^/CD117^+^ TICs in [101], we have reported that, compared to the SKOV-3 parental ovarian carcinoma cells, the highly chemoresistant subline SKOV-3-R expresses CD44, CD117 and ALDH1A, and forms spheres in stem cell medium [102]. However, these TIC-like cells also show a virtually complete loss of PGC1α expression (both as mRNA and protein) [37]. Moreover, we have not observed any HIF1α stabilization, nor any upregulation of PGC1β MYC or glucose dependence, whereas mitochondrial spare capacity is increased (work in progress). We suggest the existence of a possibly TIC-specific, PGC1α-independent alternative for maintaining OXPHOS-dependent stemness, and/or that the effects of PGC1α on the OXPHOS vs. glycolysis scale, is modified by other factors. 

### 3.2. PGC1α Autophagy, Mitophagy and Mitochondrial Dynamics

In early tumorigenesis, autophagy, or the ordered degradation and recycling of subcellular organelles in response to metabolic stress, has a tumor suppressive role, whereas in the established tumor it may allow cancer cells to survive the stress. This change in function throughout tumor progression is to some extent similar to that of PGC1α in the model presented above, but there are no or few studies that specifically address this notion. However, the inverse relationship between PGC1α and the Parkin E3 ligase, which marks out mitochondria for autophagy, is well studied in for instance neuroprotection [17] and warrants investigation in cancer, as Parkin is inactivated in several types of cancer [80]. 

Mitophagy is the specific degradation of defective mitochondria, and is thus a mitochondrial quality control system. Although mitophagy is necessary for normal tissue physiology, it is also suggested to contribute to tumor cell resistance to cellular stress and it can be part of the responses to hypoxia as well as DNA damage [103,104]. In view of the established role of PGC1α in responding to stress and in maintaining mitochondrial function and quality, we expect a coming increase in studies on PGC1α and cancer cell auto-/mitophagy. Among the few existent studies on this subject, a non-transcriptional role of PGC1α was found in MDA-MB-231 breast cancer cells, in that it bound to and stabilized the mRNA of the tumor suppressive protein mitostatin, the increased protein levels of which in turn promoted mitophagy and suppressed VEGF expression [105]. By comparison, effects on auto-/mitophagy by factors upstream of PGC1α such as AMPK, PPARγ and SIRT1, and activators thereof, such as AICAR, thiazolidinediones and resveratrol, have been extensively studied. Although this field is beyond the scope of the present review, a recent review on SIRT1 regulation of mitophagy specifically addresses activating agents such as resveratrol, particularly in the context of neuroprotection, and whether PGC1α is involved [11]; however, cancer is not discussed. 

Regulation of mitochondrial dynamics represents yet another aspect of metabolic control, and comprises two main processes: fusion of mitochondria into highly energized networks, and fission leading to dispersed mitochondria with lower membrane potential. The fission process may also precede mitophagy [106]. Fusion/fission is cell-cycle regulated but also responds to cellular stress. Regulation involves numerous proteins, notably mitofusins (MFN) 1-2 and optic atrophy 1 (OPA1) for fusion, and dynamin-related protein 1 (DRP1) and mitochondrial fission 1 (FIS1) for fission. 

Fusion/fission was found to affect migration and invasivity in breast cancer cells [107]; thus, metastatic cells showed more fission, higher DRP1 and lower MFN1 expression than non-metastatic cells. In the study on metabolism of ovarian cancer cell lines [36], both PGCα and DRP1, which was used as a mitochondrial marker, were significantly higher in the two metastatic cell lines compared to the non-metastatic. Given the several reports on increased OXPHOS in metastatic cells, increased fission in metastatic cells is counterintuitive and indicative of a need for more research on mitochondrial dynamics in cancer cells. PGCα should be included in such research, for example, to determine whether and how PGCα regulates the fusion/fission proteins. A PGCα/MFN2 axis was indicated in skeletal muscle [108], and cardiac muscle of mice deficient in PGCα–β showed altered expression of MFN1, OPA1, DRP1 and FIS1, with the PGCα/ERRα pathway directly upregulating Mfn1 during cardiac development [109]. Moreover, epoxyeicosatrienoic acids regarded as cardioprotective signaling agents were reported to act via PGC1α to increase mitochondrial biogenesis assessed as upregulation of OPA1m, MnSOD, MFN1-2 and SIRT3; this was blocked by knockdown of PGC1α [110]. To summarize, similar to auto-/mitophagy, mitochondrial dynamics are increasingly studied in cancer, and a role for PGCα in this aspect of cancer cell metabolism is suggested but remains to be explored.

## 4. PGC1α, Obesity and Cancer

In this section, we present molecular associations between obesity and cancer risk and mortality [22,111] as indications of how PGC1α could potentially be involved in tumor cell interactions with the microenvironment. Briefly, compared to the normal counterpart, the obese adipose tissue is fundamentally different, with alterations in transcription factors, miRNAs and extracellular matrix composition, and increased oxidative stress, hypoxia and infiltration of inflammatory cells creating a vicious circle of chronic inflammation [112]. The altered spectrum of growth factors, inflammatory factors and adipokines secreted by obese adipose tissue is strongly associated with cancer risk and/or mortality [22,111,113]. 

Questions of interest include whether and how the cyto-/adipokines affect tumor cell PGC1α and metabolism, and whether/how factors secreted by tumor cells affect adipocytes, and whether obese and normal adipocytes differ in these aspects. Tumors in the vicinity of adipose tissue or which metastasize to the adipocyte-rich omentum, such as breast, ovarian, renal and colon cancers, provide models for investigating these questions. Nieman et al [114] have shown that omental, rather than subcutaneous, adipocytes promoted the migration and invasion of ovarian cancer cells, and provided them with fatty acids to support growth. Similar conclusions were drawn in a study on adipocyte effects on colon cancer cells and adipocytes as well as on xenografts [115]. The same study also showed that the released fatty acids as well as exogenous oleic acid activated AMPK and survival-promoting autophagy. Although PGC1α was not examined in the two studies, their results together with previously mentioned reports on metastatic cells suggest that adipocyte-rich metastasis sites promote upregulation of PGC1α. However, in contexts of obesity or DM2, that is, in non-cancer cells, down-regulation rather than upregulation has been observed, and is often due to promoter methylation [25,68,116]. In human subjects, fatty acid overfeeding increased global methylation, and *PPARGC1A* was among the top targets [68]. Inflammatory cytokines, such as TNFα IL-6 and TWEAK, may suppress PGC1α expression in non-cancer tissue [77,78,79]. Some fatty acids are pro-inflammatory, and are associated with increased risk of colon cancer [117]. Palmitate is often used in vitro to represent pro-inflammatory fatty acids and is avidly taken up by tumor cells such as Ehrlich ascites tumor cells [113]. Interestingly, when neuronal cells as well as mouse brains were treated with palmitate, PGC1α promoter methylation increased [18]. These differences in effects on PGC1α expression are in line with cancer and non-cancer cells in general responding differently to these factors.

Adiponectin and leptin are the best studied of the adipokines of adipose tissue. Low levels of adiponectin and high levels of leptin are seen in obesity and/or DM2, and this profile is associated with increased cancer risk [111]. Adiponectin has anti-proliferative and anti-tumor effects, acts through PPAR-α (rather than PPAR-γ) and induces AMPK, SIRT1 and PGC1α and mitobiogenesis [111,118]. Leptin may also induce AMPK and activates pro-oncogenic pathways such as PI3K/Akt and JAK/STAT [111]. Leptin has shown significant stimulatory effects on growth and metabolism in cancer cell lines, for instance MCF-7 breast cancer cells, in which it induced increased expression of PGC1α and MFN2, mitobiogenesis and respiration [119]. Interestingly, DRP1 and mitophagy were also increased, and this was hypothesized to reflect accelerated cycles of mitobiogenesis and purging of damaged mitochondria that might be required in rapidly dividing cancer cells [119]. Several recent reviews, such as [120], discuss oncogenic roles of leptin, including maintenance of TIC properties, in various types of cancer. 

That adiponectin and leptin have such opposite biological effects, and yet both induce AMPK and PGC1α is in line with these proteins being orchestrators of metabolic responses. Similar to the sometimes contrary findings on PGC1α in relation to, for example, metastasis, the significance of PGC1α activation by different agents and conditions will for each case be better understood after the transcriptional activation partners of PGC1α and subsequent downstream gene activation have been elucidated and compared.

## 5. PGC1β and PGC1-Related Coactivator 

We have not touched on the other members of the PGC family, but contributions of PGC1β and PRC to the cancer cell processes discussed here must be further investigated. That PGC1β may be involved in metabolic adaptation is suggested by, for instance, lactate-induced increase in PGC1β expression and respiration in a neuroblastoma cell line [121]. Also of interest for cancer studies, PGC1β was found to mediate upregulated mitobiogenesis in response to gamma ray irradiation of colon cancer cells [122]. Hyperinsulinemia and signaling via insulin-like growth factors are of importance for cancer risk and cancer progression [111]. Insulin-like growth factor-1 (IGF-1) was found to induce PGC1β and PRC along with mitobiogenesis and mitophagy in cancer cell lines; of possible clinical significance, cells with acquired resistance to an IGF-1R inhibitor showed reduced expression of both PGC1β and PRC [123]. Of the family members, PRC is the least studied; it is involved in mitobiogenesis associated with proliferation and was also shown to be central to adaptive responses to stress [1,124]. 

## 6. Concluding Remarks

We conclude that in cancer PGC1α is neither friend nor foe, but rather the obedient servant reacting to metabolic and environmental cues to benefit the tumor cell. However, early in carcinogenesis PGC1α may be downregulated due to a protective anticancer role, whereas later in tumor progression it is often upregulated. Lipogenesis and fatty acid metabolism are particularly often found to be upregulated in tumor cells with high expression of PGC1α Overall, PGC1α contributes to a tumor cell phenotype with a capacity for metabolic plasticity, that is, an ability to utilize at short notice many different types of nutrients, and to thereby adapt to cellular stress [36,92,101]. This metabolic potential has been observed among proliferating as well as migrating tumor bulk cells as well as among TICs, but how the potential is fine-tuned in response to specific cues needs further investigation. It is thus not known exactly how the overall metabolic plasticity of tumor cells is connected to chemoresistance and the process of metastasis, but with its capacity for rapid responses and numerous downstream pathways PGC1α can clearly play several parts therein. This responsiveness to, or dependence on, microenvironmental cues may turn out to explain why PGC1α levels are high or low in specific cancers, at least in part. With bearing on fatty acid metabolism, we have also mentioned here the influence of adipose tissue tumor microenvironments on tumor cell metabolism—another field of inquiry where the effects on PGC1α activity and function have not been sufficiently addressed. 

Cancer cells differ from normal cells in many known and unknown ways. The capabilities and possibilities of PGC1α are likely similar in cancer and normal cells. The question is how the cancer cell makes use of them. We believe that the various coactivation partners—in addition to ERRα—and downstream gene expression networks of PGC1α need to be examined in order to better understand the fine-tuned, and possibly tissue- and/or clone-dependent, specifics of PGC1α-mediated effects. While PGC1α itself is likely not suitable for pharmacological targeting, its downstream effectors may be; inhibiting a crucial factor therein might conceivably render the cancer cell unable to modulate its metabolism and thereby sensitize it to other treatment.
